# Design and Characterization of a Self-Oscillating Fluxgate-Based Current Sensor for DC Distribution System Applications

**DOI:** 10.3390/s25082360

**Published:** 2025-04-08

**Authors:** Wei Chen, Huaijie Chen, Haibo Xu, Li Li

**Affiliations:** 1 The Key Laboratory of Smart Low-Voltage Apparatus and New Energy Application of Zhejiang Province, Wenzhou University, Wenzhou 325035, China; chenwei1989@wzu.edu.cn (W.C.);; 2 School of Mechanical Engineering, Zhejiang University, Hangzhou 310007, China; 3 Zhejiang CHINT Electrics Co., Ltd., Wenzhou 325600, China

**Keywords:** current sensor, distribution system, noise suppression, self-oscillating fluxgate

## Abstract

Fluxgate-based current sensors are usually implemented for DC current detection, but their complex structure and circuits with large volume and high cost have been limiting their applications. This paper presents a low-cost sensor with a one-core-three-winding structure that can be suitable for integrated measurement in distribution system applications. Based on a self-oscillating scheme, the new sensor introduces an induction winding to suppress the noise caused by the transformer effect instead of adding more magnetic cores. The transmission and transfer functions of the sensor, based on nonlinear magnetization, are conducted for the qualitative and quantitative analysis. A prototype is fabricated and several specifications including linearity, small-signal bandwidth, output noise, and power-on repeatability are characterized. Experimental results show that the proposed sensor realizes an accuracy better than 0.15% with a range of 0–600 A. By implementing the proposed noise suppression method, the signal-to-ratio is improved from 19.55 dB to 48.88 dB. Compared with a traditional fluxgate sensor with a three-core-four-winding structure, the proposed sensor reduces the volume by 44.4% and the cost by 23.6%, indicating a good prospect for practical applications.

## 1. Introduction

With the rapid increase in the installed capacity of new energy power generation, addressing the issue of new energy consumption has become a pressing concern for the power system [1]. In response, DC distribution systems have emerged as a crucial development trend in modern distribution systems due to their ability to efficiently and flexibly accommodate distributed generation. Furthermore, the growing demand for computing power in artificial intelligence applications has led to an increased need for internet data centers, which can also benefit from DC distribution systems due to their 10–20% lower power costs than AC systems. The protection and control of DC distribution systems rely on electrical appliances that operate based on accurate current detection strategies. In this study, the authors have investigated several commonly used DC current detection methods, including shunt, Hall effect, giant magnetoresistance, tunnel magnetoresistance, magneto-optical, and fluxgate [2,3,4,5,6,7]. The features of these different current detection methods, such as bandwidth, accuracy, temperature drift, range, and power dissipation, are discussed in Table 1. Among these methods, the fluxgate method has garnered significant attention due to its high accuracy, low temperature drift, and low power dissipation.

**Figure 1:**
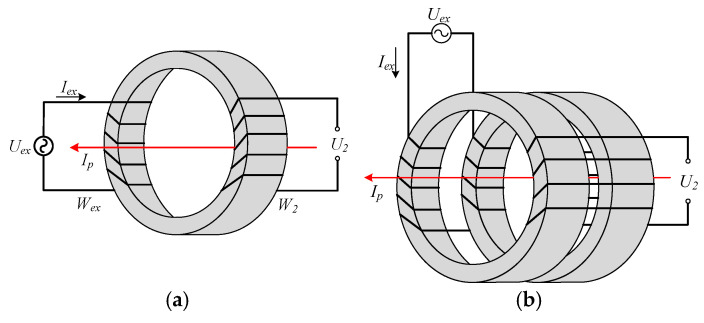
Scheme of the magnetic modulation current probes. (**a**) Traditional magnetic modulation current probe. (**b**) Dual-core magnetic modulation current probe.

Previous research has established that fluxgate-based sensors are quite suitable for high-precision detection and power system applications [8]. A traditional magnetic modulation DC sensor probe, shown in Figure 1a, realizes the DC current detecting by the second harmonic. It has previously been observed that the amplitudes of even harmonics are proportional to the magnitude of the primary current *Ip* [9]. Therefore, the magnitude and the direction of *Ip* could be detected by obtaining second-harmonic parameters through specific demodulation circuits. Evidence indicates that the rather small even harmonic amplitudes make it easy to be submerged in the noise caused by the transformer effect, resulting in a low signal-to-noise ratio (SNR) [10]. Therefore, several attempts have been made to improve the SNR by implementing dual-toroidal magnetic cores, as shown in Figure 1b. The exciting current induces magnetic fluxes of the same magnitude and the opposite direction in the dual toroidal cores. In this way, the odd harmonics in the inductive voltage are neutralized, while the even ones are superposed to improve the SNR [11]. However, the dual-core structure requires more installation space along with complicated modulation circuits. Furthermore, accuracy loss from the inconsistency of the two cores is inevitable [12].

Some studies provided another magnetic modulation method, in which the magnetic core with DC bias magnetic flux is treated as a non-linear impedance in a self-oscillating circuit [12,13,14,15]. The self-oscillating fluxgate method has been widely used to reduce complexity and cost while keeping the advantages of traditional fluxgate sensors [16]. In this way, the dismissal of the exciting supply contributes to the decrease of both power dissipation and volume. The authors in the literature [17,18] found that the exciting current’s average value and duty ratio are proportional to the primary current when the parameters of the magnetic core and the oscillating circuit components are cautiously designed. Extensive research has been carried out to improve the range, accuracy, stability, and SNR for metrological calibration and on-site measurement [14,19]. High accuracy, wide range, or high stability always come with the sacrifice of volume [20]. However, the installation space is valuable in appliances for distribution systems. Few works focus on the realization of a relatively high precision measurement within a limited volume for integrated applications.

This article presents a low-cost self-oscillating fluxgate-based current sensor with a one-core-three-winding structure and relatively simple circuits for DC distribution system applications. Firstly, the operating principle, mathematical model, and quantitative analysis of the transformer based on nonlinear magnetization are introduced. Then, the design of the magnetic components and circuits is presented. As verification, an example prototype for a specific appliance is realized and several specifications are characterized. Finally, comparison results between the proposed sensor and a multi-core fluxgate sensor are given.

## 2. Methodology

**Figure 2:**
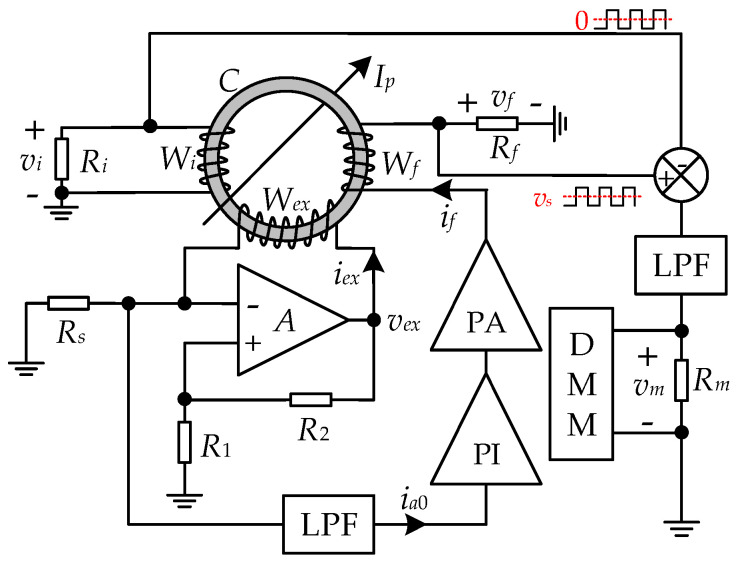
Diagram of the proposed self-oscillating fluxgate-based current sensor.

The diagram of the sensing system is shown in Figure 2. The sensor is composed of a sensor probe, a self-oscillating circuit, a feedback circuit, and a noise suppression circuit. The sensor probe contains a magnetic toroidal core wound with an excitation winding *W_ex_* with *N_ex_* turns, a feedback winding *W_f_* with *N_f_* turns, and an induction winding *W_i_* with *N_i_* turns. The primary current *I_p_* flows through the magnetic core via a single turn (*N_p_* = 1) conductor. *W_ex_*, along with a voltage comparator, a sampling resistor *R_s_*, and two threshold resistors *R*_1_ and *R*_2_, form the self-oscillating circuit, generating alternating square waves to saturate the core periodically. Coupled with the DC flux by *I_p_*, the non-linear alternating magnetic flux turns *W_ex_* into a variable nonlinear inductor. The feedback circuit consists of a low pass filter (LPF), a proportional-integral (PI) control circuit, a power amplifier (PA), *W_f_*, and a feedback resistor *R_f_*. The noise suppression circuit is formed by *W_i_*, a noise sampling resistor *R_i_*, a subtraction circuit, an LPF, and an output sampling resistor *R_m_*. The voltage *v_m_* on the potential terminal of *R_m_* is measured by a digital multimeter (DMM) as the output signal of the proposed sensor.

### 2.1. Measurement Principle

**Figure 3:**
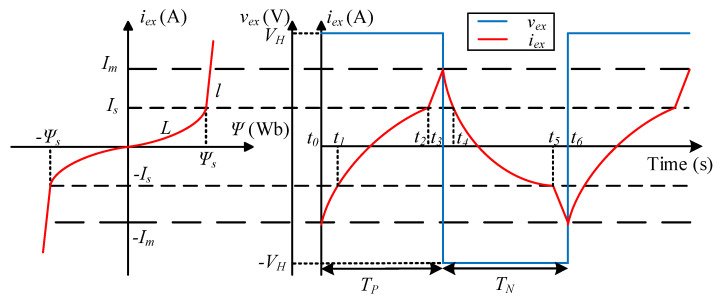
Waveforms of the self-exciting fluxgate circuit when the primary current is nonzero. Waveforms on the left are the nonlinear magnetic flux, while the ones on the right are the exciting voltage and current.

The differential equation of the self-oscillating fluxgate circuit is expressed as follows:

(1) $$\left(R_{s}+R_{c}\right)i_{ex}\left(t\right)+L\frac{di_{ex}\left(t\right)}{dt}=v_{ex}\left(t\right)$$

where *R_s_* is the sampling resistance, and *R_c_*, *i_ex_*, *L*, and *v_ex_* are the internal resistance, the current, the inductance, and the voltage of the exciting winding *W_ex_*, respectively. The non-linear magnetic flux and the exciting voltage and current waves are shown in Figure 3. It can be observed that the rising exciting current passes through the reverse saturation region, unsaturation region, and forward saturation region within the *T_P_* interval. Since the time constants for the saturation and the unsaturation regions are different, the *T_P_* interval is divided into three intervals.

**Figure 4:**
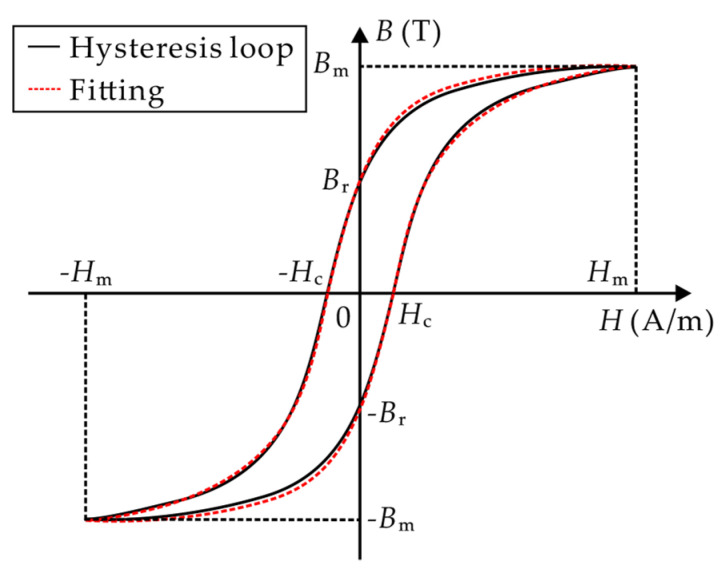
Hysteresis loop and its arctangent fitting curve.

M. Ponjavic and R. M. Duric from Belgrade University were the first to establish an average current model based on the arctangent function model of the magnetization curve [16]. To obtain a more accurate fitting function of the magnetization curve, this paper introduces the hysteresis effect based on the arctangent fitting by Equation (2), as shown in Figure 4.

(2) $$B\left(H\right)=\{\begin{array}{ll} a\arctan\left[b\left(H-H_{c}\right)\right] & t\in \left[0,T_{P}\right]\\ a\arctan\left[b\left(H+H_{c}\right)\right] & t\in \left[T_{P},T_{P}+T_{N}\right] \end{array}$$

In Equation (2), the algebraic symbols $a=2B_{s}/\pi$ and $b=\pi \mu_{0}\mu_{m}/\left(2B_{s}\right)$. *B* is the magnetic flux density, *H* is the magnetic field intensity, and *H_c_* is the coercivity parameter. *T_P_* and *T_N_* are the time intervals for the rising and falling exciting currents, respectively. *μ*_0_ is the permeability of the vacuum, *μ_m_* is the maximum (Max.) magnetic permeability of the magnetic core, and *B_s_* is the saturation magnetic flux density. According to Ampere’s law, Equation (2) can be rewritten to obtain the time function of the flux density.

(3) $$B\left(t\right)=\{\begin{array}{ll} a\arctan\frac{b\left[N_{ex}\left(i_{ex}\left(t\right)+nI_{p}\right)-H_{c}l_{c}\right]}{l_{c}} & t\in \left[0,T_{P}\right]\\ a\arctan\frac{b\left[N_{ex}\left(i_{ex}\left(t\right)+nI_{p}\right)+H_{c}l_{c}\right]}{l_{c}} & t\in \left[T_{P},T_{P}+T_{N}\right] \end{array}$$

where *N_ex_* is the number of the exciting winding *W_ex_*, *n* is the ratio of the primary winding number *N_p_* to *N_ex_*, and *l_c_* is the effective magnetic length. Thus, the variable inductance *L*(*t*) can be calculated by the magnetic flux *ψ* as follows:

(4) $$L\left(t\right)=\frac{d\psi \left(t\right)}{di_{ex}\left(t\right)}=\{\begin{array}{ll} \frac{C}{1+D^{2}{\left[i_{ex}\left(t\right)+I_{ad}^{+}\right]}^{2}} & t\in \left[0,T_{P}\right]\\ \frac{C}{1+D^{2}{\left[i_{ex}\left(t\right)+I_{ad}^{-}\right]}^{2}} & t\in \left[T_{P},T_{P}+T_{N}\right] \end{array}$$

where the algebraic symbols $I_{ad}^{+}=nI_{p}-H_{c}/N_{ex}$, $I_{ad}^{-}=nI_{p}+H_{c}/N_{ex}$, $C=N_{ex}^{2}S\mu_{0}\mu_{m}/l_{c}$, and $D=bN_{ex}/l_{c}$. *S* is the cross-sectional area of the magnetic core. It is hard to directly calculate the average value of the exciting current *i_ex_* by calculating the indefinite integral using Equations (1) and (4). Therefore, this paper uses the average voltage *V_ex_* to derive the expression of *I_ex_*.

(5) $$V_{ex}=\frac{\int_{0}^{T_{P}+T_{N}}v_{ex}\left(t\right)dt}{T_{P}+T_{N}}=\frac{\int_{0}^{T_{P}+T_{N}}\left[v_{R}\left(t\right)+v_{L}\left(t\right)\right]dt}{T_{P}+T_{N}}$$

where *v_R_* is the sum of the voltage of *R_s_* and *R_c_*, and *v_L_* is the voltage of *L*. According to the waveforms in Figure 4, the exciting voltage *v_ex_* in one cycle only equals $\pm V_{H}$. Thus, the average voltage *V_ex_* can be calculated as follows:

(6) $$V_{ex}=\frac{T_{P}-T_{N}}{T_{P}+T_{N}}V_{H}$$

where *V_H_* is the amplitude of the exciting voltage. The average value of *i_ex_* can calculated as follows:

(7) $$I_{ex}=\frac{V_{ex}}{R_{s}+R_{c}}=\frac{T_{P}-T_{N}}{T_{P}+T_{N}}I_{H}$$

where *I_ex_* is the average value of *i_ex_*, and *I_H_* is the steady-state current under *V_H_*. According to Figure 4, the values of the exciting current *i_ex_* at the time of 0, *T_P_*, *T_P_* + *T_N_* are expressed as follows:

(8) $$i_{ex}\left(0\right)=i_{ex}\left(T_{P}+T_{N}\right)=-I_{m},\;i_{ex}\left(T_{P}\right)=I_{m}$$

where *I_m_* is the peak value of the exciting current. To solve the differential equation, Equation (1) is converted as follows:

(9) $$\{\begin{array}{ll} \frac{L\left(i_{ex}\right)}{\left(I_{H}-e_{ex}\right)}di_{ex}=dt & t\in \left[0,T_{P}\right]\\ \frac{L\left(i_{ex}\right)}{\left(-I_{H}-e_{ex}\right)}di_{ex}=dt & t\in \left[T_{P},T_{P}+T_{N}\right] \end{array}$$

Combined with Equation (8), the definite integral of Equation (9) is derived as follows:

(10) $$\{\begin{array}{l} \int_{-I_{m}}^{I_{m}}\frac{L\left(i_{ex}\right)}{\left(I_{H}-e_{ex}\right)}di_{ex}=\int_{0}^{T_{P}}dt\\ \int_{I_{m}}^{-I_{m}}\frac{L\left(i_{ex}\right)}{\left(-I_{H}-e_{ex}\right)}di_{ex}=\int_{T_{P}}^{T_{P}+T_{N}}dt \end{array}$$

By substituting Equation (4) into Equation (10), the intervals *T_P_* and *T_N_* are obtained as follows:

(11) $$\{\begin{array}{l} T_{P}=C\frac{2DE_{ad}^{+}\left(I_{H}+I_{ad}^{+}\right)+\ln\frac{F_{ad}^{+}{\left(I_{H}+I_{m}\right)}^{2}}{{\left(I_{H}-I_{m}\right)}^{2}}}{2D^{2}{\left(I_{H}+I_{ad}^{+}\right)}^{2}+1}\\ T_{N}=C\frac{2DE_{ad}^{-}\left(I_{H}-I_{ad}^{-}\right)+\ln\frac{F_{ad}^{-}{\left(I_{H}+I_{m}\right)}^{2}}{{\left(I_{H}-I_{m}\right)}^{2}}}{2D^{2}{\left(I_{H}-I_{ad}^{-}\right)}^{2}+1} \end{array}$$

where the algebraic symbols are presented in Equation (12).

(12) $$\{\begin{array}{l} E_{ad}^{+}=\arctan\left[D\left(I_{ad}^{+}+I_{m}\right)\right]-\arctan\left[D\left(I_{ad}^{+}-I_{m}\right)\right]\\ E_{ad}^{-}=\arctan\left[D\left(I_{ad}^{-}+I_{m}\right)\right]-\arctan\left[D\left(I_{ad}^{-}-I_{m}\right)\right]\\ F_{ad}^{+}=\frac{D^{2}{\left(I_{ad}^{+}+I_{m}\right)}^{2}+1}{D^{2}{\left(I_{ad}^{+}-I_{m}\right)}^{2}+1}\\ F_{ad}^{-}=\frac{D^{2}{\left(I_{ad}^{-}+I_{m}\right)}^{2}+1}{D^{2}{\left(I_{ad}^{-}-I_{m}\right)}^{2}+1} \end{array}$$

In the sensor’s practical design, *R_s_*, *R_c_,* and *n* are usually rather small, resulting in a relation between *I_H_*, the exciting current peak value *I_m_*, and the primary current *I_p_*.

(13) $$I_{H}\gg I_{m}\gg nI_{p}$$

According to Equation (13), the logarithmic terms in Equation (11) are approximately equal to zero. When the conditions in Equation (13) hold, Equation (7) can be simplified by substituting Equation (11).

(14) $$I_{ex}=nI_{p}\frac{-2D^{2}+2D^{2}{\left(\frac{nI_{p}}{I_{H}}\right)}^{2}+\frac{1}{I_{H}^{2}}}{2D^{2}-2D^{2}{\left(\frac{nI_{p}}{I_{H}}\right)}^{2}+\frac{1}{I_{H}^{2}}}$$

When D≫1, the terms with *I_H_* as the denominator are approximately equal to zero, deriving a linear model between the exciting current *I_ex_* and the primary current *I_p_*.

(15) $$I_{ex}=-nI_{p}$$

The linear model is based on the hypothesis of $I_{H}\gg I_{m}\gg nI_{p}$ and D≫1, which indicates that higher Max. magnetic permeability *μ_m_*, lower saturation magnetic flux density *B_s_*, and lower coercivity *H_c_* are beneficial to improve the linearity. In the closed-loop system, the self-oscillating circuit detects the DC bias magnetic balance inside the magnetic core and outputs *i_a_*_0_, which characterizes the magnetic balance condition. The feedback circuit generates feedback current to counteract the magnetic bias. When the magnetic balance is achieved in ideal conditions, *I_p_* can be measured by *i_f_*.

(16) $$I_{p}=\frac{N_{f}}{N_{p}}i_{f}$$

where *i_f_* is the current of the feedback winding.

### 2.2. Analysis of the Transformer Noise and Its Suppression

In a closed-loop system, especially in the architecture of a single magnetic core, the noise caused by the transformer effect is more pronounced. Since *W_f_* and *W_ex_* are wound on the same magnetic core, the magnetic flux from the exciting current induces a voltage *E_f_* in *W_f_*.

(17) $$E_{f}=-N_{f}S\frac{dB\left(t\right)}{dt}=-kN_{f}S\mu_{0}\mu_{m}\frac{di_{ex}\left(t\right)}{dt}$$

where *N_f_* is number of the feedback winding, and coefficient *k* is related to the width and thickness of the magnetic core. Assuming the magnetic balance condition is achieved, *i_ex_* can be obtained as follows:

(18) $$i_{ex}\left(t\right)=\{\begin{array}{ll} I_{H}-\left(I_{H}+i_{s}\right)e^{\frac{-t}{\tau }} & 0\leq t<T_{P}\\ \left(I_{H}+i_{s}\right)e^{\frac{T_{P}-t}{\tau }}-I_{H} & T_{P}\leq t<T \end{array}$$

where *i_s_* is the current corresponding to the saturation knee point of the magnetic core, *τ* is the time constant of the exciting circuit, and $T_{P}=T_{N}=\tau \ln\frac{I_{H}+i_{s}}{I_{H}-i_{s}}$. By substituting Equation (18) to Equation (17), it gives the following:

(19) $$E_{f}\left(t\right)=\{\begin{array}{ll} -\frac{kN_{f}S\mu_{0}\mu_{m}}{\tau }\left(I_{H}+i_{s}\right)e^{\frac{-t}{\tau }} & 0\leq t<T_{P}\\ \frac{kN_{f}S\mu_{0}\mu_{m}}{\tau }\left(I_{H}+i_{s}\right)e^{\frac{T_{P}-t}{\tau }} & T_{P}\leq t<T \end{array}$$

**Figure 5:**
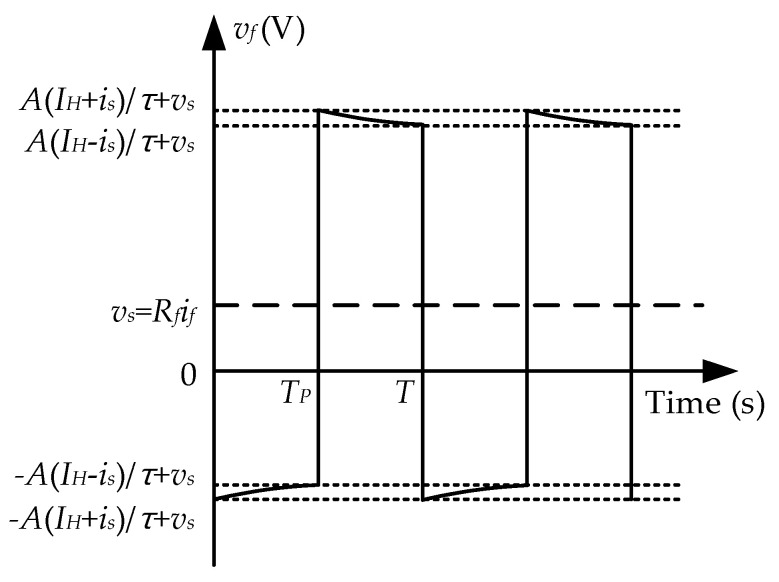
Waveforms of *v_f_* at the feedback resistor with $A=kN_{f}S\mu_{0}\mu_{m}$.

The voltage *v_f_* is the sum of *E_f_* and the feedback voltage *v_s_* at *R_f_*, resulting in a similar square wave oscillating up and down with *v_s_* as the reference value, as shown in Figure 5. Due to the presence of noise, the SNR of the output signal *v_m_* is rather low. To suppress the noise caused by the transformer effect, we introduce an induction winding *W_i_* instead of a signal filter. The turn number of *W_i_* is consistent with one of the feedback winding *W_f_* to obtain an induced voltage *v_i_*, which shares the same amplitude and phase as *E_f_*. The noise is suppressed by implementing a subtraction operation circuit for *v_i_* and *E_f_*, leaving only v_s_. In practice, it is difficult for *v_i_* and *E_f_* to achieve complete amplitude and phase consistency. Therefore, an LPF circuit is utilized to help eliminate the residual noise and obtain a smooth voltage waveform of *v_m_*.

### 2.3. Derivation of the Transfer Function and Steady Error

**Figure 6:**
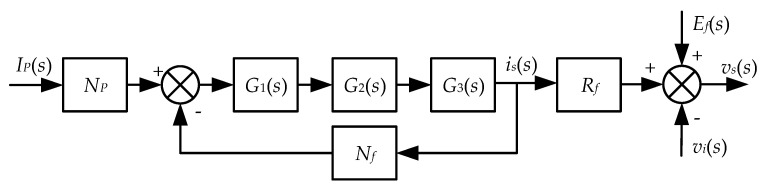
Control block diagram of the closed-loop system.

During the derivation of the transfer function of the closed-loop system, some errors in the system are idealized to simplify the model. Firstly, the hysteresis loss, eddy current loss, and leakage flux are ignored. Secondly, we assume that the LPFs, compactors, and PA are ideal for neglecting their influence on DC circuits. Finally, the parasitic capacitances inside the windings and in the printed circuit board layout are not considered, ignoring the capacitive error of the system. Based on the above assumptions, the block diagram of the closed-loop system is obtained, as shown in Figure 6.

*E_f_*(s), including a transformer model composed of *W_ex_*, *W_f_*, and the magnetic core, is the noise caused by the transformer effect on *W_f_*. *E_f_*(s) is represented by the following equation.

(20) $$E_{f}\left(s\right)=\frac{N_{f}}{N_{ex}}$$

Here $N_{i}=N_{s}$ and $R_{3}=R_{4}$, leading to a cancel out of *E_i_*(*s*) and *E_f_*(*s*). In this way, only the transfer function between *I_p_* and *i_f_* is considered in the closed-loop system.

(21) $$G\left(s\right)=\frac{i_{f}\left(s\right)}{I_{p}\left(s\right)}=\frac{N_{p}G_{1}\left(s\right)G_{2}\left(s\right)G_{3}\left(s\right)}{1+N_{f}G_{1}\left(s\right)G_{2}\left(s\right)G_{3}\left(s\right)}=\frac{N_{p}}{N_{f}}\left(1-\frac{1}{1+N_{f}G_{1}\left(s\right)G_{2}\left(s\right)G_{3}}\right)$$

where *G*_1_(*s*), *G*_2_(*s*), and *G*_3_(*s*) are the transfer functions of the self-oscillating circuit, the PI and PA circuits, and the feedback circuit, respectively.

(22) $$\{\begin{array}{l} G_{1}\left(s\right)=-\frac{R_{s}}{N_{ex}}\\ G_{2}\left(s\right)=K_{\mathrm{PI}}K_{\mathrm{PA}}\left(1+\frac{1}{j\omega \tau_{\mathrm{PI}}}\right)\\ G_{3}\left(s\right)=\frac{1}{R_{m}+Z_{s}}=\frac{l_{c}}{R_{m}l_{c}+j\omega \mu_{0}\mu_{m}SN_{f}^{2}} \end{array}$$

where *K*_PI_ is the proportional factor of the PI circuit, *K*_PA_ is the gain of the PA circuit, *ω* is the radian frequency, *τ*_PI_ is the time constant of the PI circuit, *R_m_* is the output sampling resistance, and *Z_s_* is the complex impedance of the feedback winding *W_f_*. The closed-loop system reaches a steady state in the condition that the magnetic flux by the feedback current exactly offsets the one by the primary current *I_p_*. The transfer function in the steady state is $G\left(s\right)=N_{p}/N_{f}$. Compared to Equation (21), the steady error of the closed-loop system *σ* is conducted as follows:

(23) $$\sigma \left(s\right)=-\frac{1}{1+N_{f}G_{1}\left(s\right)G_{2}\left(s\right)G_{3}\left(s\right)}=-\frac{1}{1-\frac{K_{\mathrm{PI}}K_{\mathrm{PA}}N_{f}R_{s}l_{c}}{N_{ex}\left(l_{c}R_{m}+j\omega \mu_{0}\mu_{m}SN_{f}^{2}\right)}\left(1+\frac{1}{j\omega \tau_{\mathrm{PI}}}\right)}$$

When the primary current *I_p_* is a pure DC current, the current frequency is 0 Hz, indicating that *jω* equals 0. Thus, Equation (23) is rewritten as follows:

(24) $$\sigma \left(s\right)=-\frac{1}{1-\frac{K_{PI}K_{PA}N_{f}R_{s}}{N_{ex}R_{m}}\left(1+\infty \right)}\approx 0$$

Equation (24) indicates that the closed-loop system is an error-free system under the previously mentioned assumptions. The theoretical error that approaches 0 is mainly attributed to the PI circuit, whose gain approaches infinity when the input is a DC signal. However, an infinite gain is impossible in practical applications, resulting in an error caused by the open-loop gain. In addition, Equation (24) indicates that increasing the number of *N_f_* and *R_s_*, and decreasing the number of *N_ex_* and *R_m_*, can also reduce the steady-state error of the closed-loop system.

## 3. Design and Realization

Based on the methodology in Section 2, this section illustrates the characteristics analysis and design criteria of the proposed current sensor for DC distribution system applications. An example prototype was designed for a DC molded case circuit breaker (MCCB) from CHINT company (Wenzhou, China) with a rated current of 400 A, whose inner installation space for each phase is 48 × 48 × 30 mm (length × width × height). Considering the measurement in normal and 1.5 times overload operations, the current sensor is expected to achieve a higher accuracy than 0.5% in a range of 0 to 600 A.

### 3.1. Magnetic Core and Windings

According to Equation (11), the design parameters that affect the sensor performance can be divided into magnetic and circuit parameters. The magnetic parameters include the Max. magnetic permeability *μ_m_*, saturation magnetic flux density *B_s_*, coercivity *H_c_*, effective magnetic path length *l_c_*, magnetic core cross-sectional area *S*, and exciting winding turn number *N_ex_*. The authors found that the proposed sensor gains a higher accuracy with larger *μ_m_* and *N_ex_*, and smaller *B_s_*, *H_c_*, and *l_c_*, while the influence of *S* on the system performance is negligible. According to Equation (16), it can be inferred that increasing the turn number *N_f_* of the feedback winding *W_f_* leads to an increase in the measurement range of the closed-loop system. However, the inductance of *W_f_* increases by the square of *N_f_*, challenging the driving capability of the PA circuit. Similarly, the time constant of the system also increases with *N_f_*, resulting in a worse dynamic responsibility. Furthermore, an increase in *N_f_* will enlarge the noise caused by the transformer effect, which is proportional to *N_f_* in Equation (19). The Fe-base Nanocrystalline Alloy in model 1K107, with high Max. magnetic permeability, low saturation magnetic flux density, and low coercivity, is selected for the magnetic core. Taking the current detection range and the installation limits of the MCCB as constraint conditions, the parameters of the magnetic core and the windings are designed and optimized by electromagnetic and circuit simulations combined with a genetic algorithm. The magnetic parameters are listed in Table 2.

### 3.2. Circuit Design

**Figure 7:**
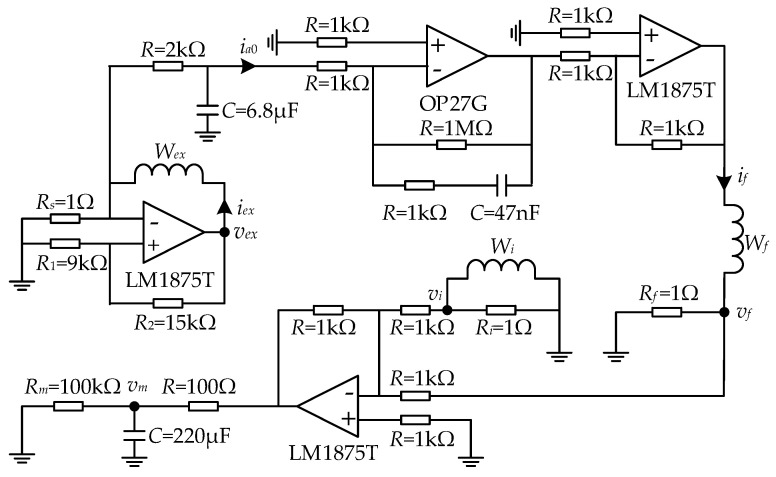
Circuit scheme of the proposed current sensor.

The scheme of the analog circuit of the proposed current sensor is shown in Figure 7. The exciting winding *W_ex_* is excited by the self-oscillating circuit, whose square voltage is realized by a power amplifier chip (LM1875T). To ensure the normal operation of the exciting square voltage generation, the voltage drop on *R_s_* by the steady current *I_H_* must be larger than the one on *R*_1_. The constraint is expressed as follows:

(25) $$\frac{R_{s}}{R_{s}+R_{c}}\leq \frac{R_{1}}{R_{1}+R_{2}}$$

where *R*_1_ and *R*_2_ are the threshold resistances. The right-side term of Equation (21) is denoted as the flip voltage ratio, which is a monotonic positive correlative to the accuracy and range of the proposed sensor.

The cut-off frequency of the LPF connected to *W_ex_* is designed as 11.70 Hz with a simple RC circuit, while the oscillating frequency is designed as 270 Hz. Amplifier OP27G, with a high slew rate of 2.8 V/μs and a wide gain bandwidth of 8 MHz, is selected for the PI circuit because of the demand for rapid voltage flipping. The proportionality coefficient *K*_PI_ and the integral time are 1 and 4.7 μs. The selection of small values of *K*_PI_ and integral time are beneficial to improve loop stability and expand bandwidth at high frequencies, respectively. The PA circuit with a gain of −1 is built to supply a sufficiently large feedback current achieving the magnetic balance inside the magnetic core. Since the resistor in the LPF of the noise suppression circuit will participate in voltage distribution, the ratio of *R_m_* to the resistor in the LPF should be large enough to improve the sampling accuracy. Simulation at a current of 250 A was carried out in the NI (Austin, TX, USA) Multisim software V14 to verify the circuit design.

**Figure 8:**
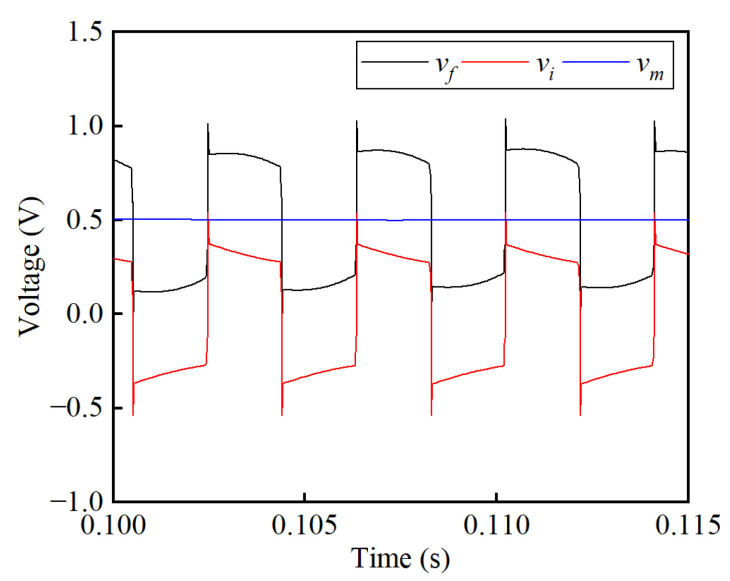
Simulation results of the induced and output voltage before and after noise suppression at the primary current of 250 A.

**Figure 9:**
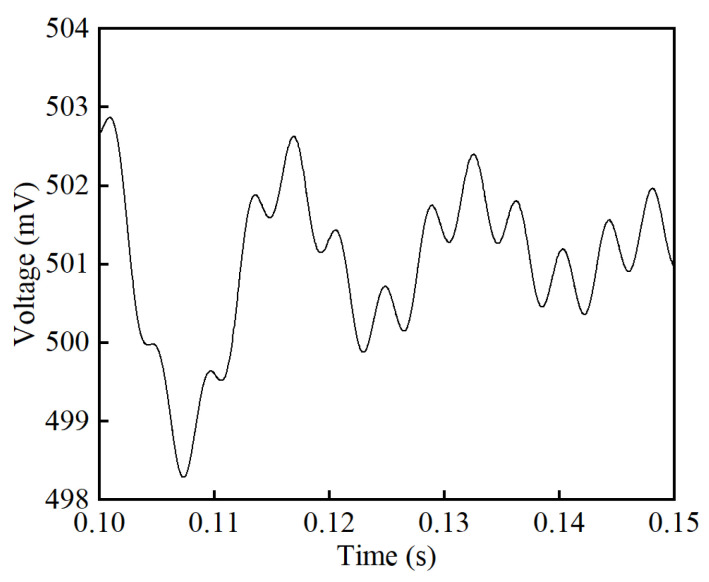
Simulation results of the ripple voltage in *v_m_* at the primary current of 250 A.

The effect of the noise suppression circuit is presented in Figure 8. Without the noise suppression circuit, the sensor’s output voltage is measured on the feedback resistor *R_f_*, marked as *v_f_*. As expected, the voltage *v_f_* in Figure 8 is consistent with the frequency of the exciting voltage square wave. The signal *v_f_* is an oscillating wave with an average value of 0.5 V, while the SNR is 3.35 dB. This measured signal is averaged, transferred to a current value, and multiplied *N_f_* times to obtain the primary current *I_p_*. With the noise suppression circuit, the induced voltage *v_i_* generated on the induction winding *W_i_* is conducted to cancel out the oscillating square wave in *v_f_*. Regardless of the high-order harmonics, signal *v_i_* is consistent with the amplitude and phase of signal *v_f_*, resulting in a rather smooth DC signal *v_m_* after subtraction and filtering. By extracting a segment from the *v_m_* waveforms, the ripple voltage caused by voltage fluctuations is under scrutiny, as shown in Figure 9. Analysis shows that the peak-to-peak value of ripple voltage in *v_m_* is 4.33 mV, giving an SNR of 45.61 dB. Results show that a significant improvement is achieved by implementing the noise suppression circuit.

### 3.3. Finished Prototype

**Figure 10:**
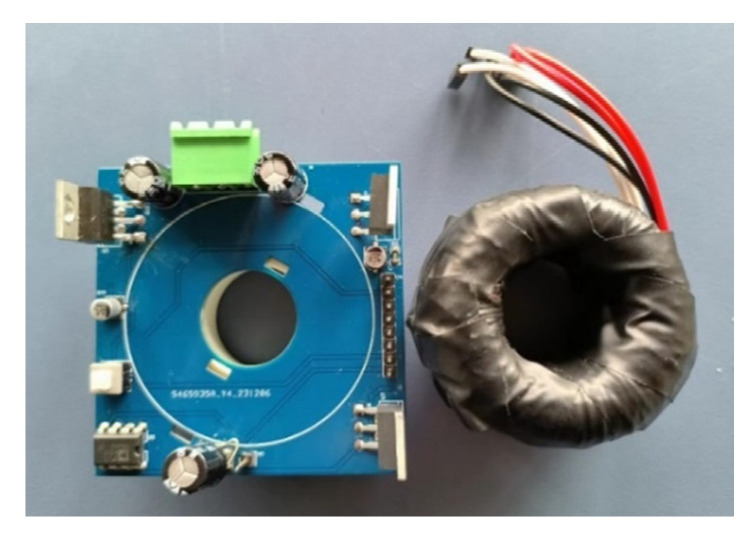
Photograph of the prototype sensor.

A prototype based on the designed single-core-three-winding sensor is fabricated and shown in Figure 10. The dimensions of the sensor probe are 43.81 × 23.37 × 20.54 mm in external diameter, internal diameter, and height, which can be installed in CHINT’s MCCBs with a rated current of 400 A.

## 4. Experimental Results and Discussion

**Figure 11:**
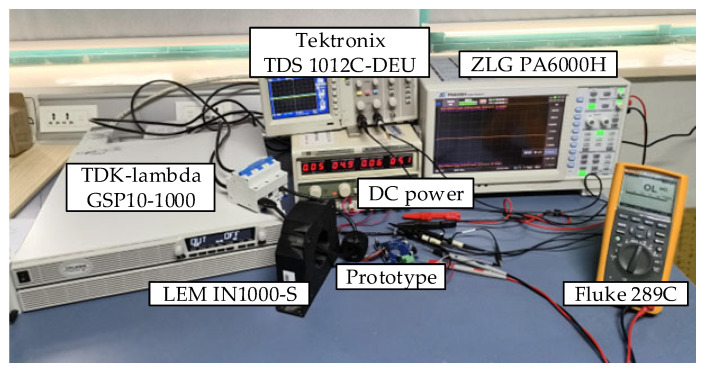
Experiment setup.

The experiment setup is shown in Figure 11. The primary current *I_p_* is generated by a TDK-lambda (Tokyo, Japan) GSP10-1000 power supply with a rated current output of 1000 A, a Max. line regulation of 0.05% at the rated current, and a temperature stability of 0.01%. The actual value of *I_p_* is precisely measured by a LEM (Geneva, Switzerland) IN1000-S current sensor with an accuracy of 0.0018% and a ZLG (Guangzhou, China) PA6000H power analyzer with an accuracy of 0.01%. The voltage and current waves are observed by a Tektronix (Beaverton, OR, USA) TDS 1012C-DEU oscilloscope. The voltage root mean square (RMS) on the potential terminal of the output sampling resistor *R_m_* is measured by a Fluke (Everett, WA, USA) 289C with an accuracy of 0.025%. The voltage RMS is then divided by n to obtain the measured current of the proposed current sensor. To evaluate the performance of the proposed sensor, specifications including linearity, small-signal bandwidth, output noise, and power-on repeatability are characterized. Furthermore, the characteristics of the proposed sensor are compared with other sensors.

### 4.1. Linearity

**Figure 12:**
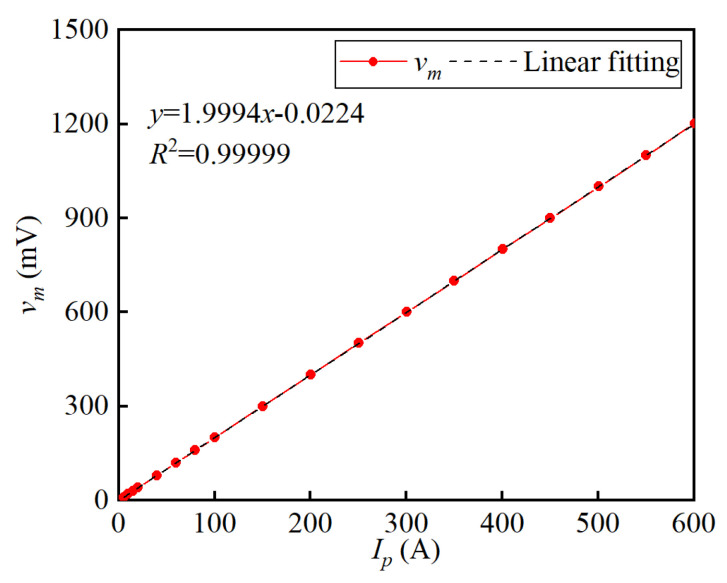
Relationship between the output voltage *v_m_* and the primary current *I_p_*.

The linearity is measured by varying the primary current from 0 A to 20 A at 5 A intervals, from 20 A to 100 A at 20 A intervals, and from 100 A to 600 A at 50 A intervals. Figure 12 shows the experimental verified linear dependence between *v_m_* and *I_p_*. The linear fitting equation indicates that the sensitivity of the proposed current sensor is 1.9994 mV/A, which is very close to the theoretical value of 2 mV/A calculated by $S_{v}=nR_{m}$. When the primary current *I_p_* is 600 A, the output signal *v_m_* is 1201.50 mV. The Max. linear absolute error value is 0.837 mV, indicating that the linearity of the prototype is 0.07%.

**Figure 13:**
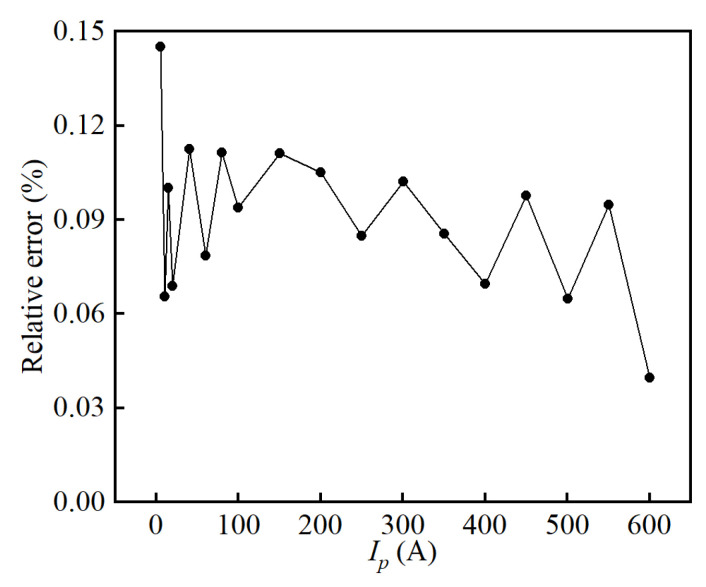
Relative errors of the proposed sensor in the range of 0–600 A.

Figure 13 shows the relative errors of the prototype. It shows that the relative errors of the designed sensor are below 0.15%, while the Max. relative error is discovered when the primary current is as small as 5 A as 0.83% of the full range due to the influences of errors from, e.g., parasitic capacitance, zero offset, unideal gain, and so on.

### 4.2. Small-Signal Bandwidth

**Figure 14:**
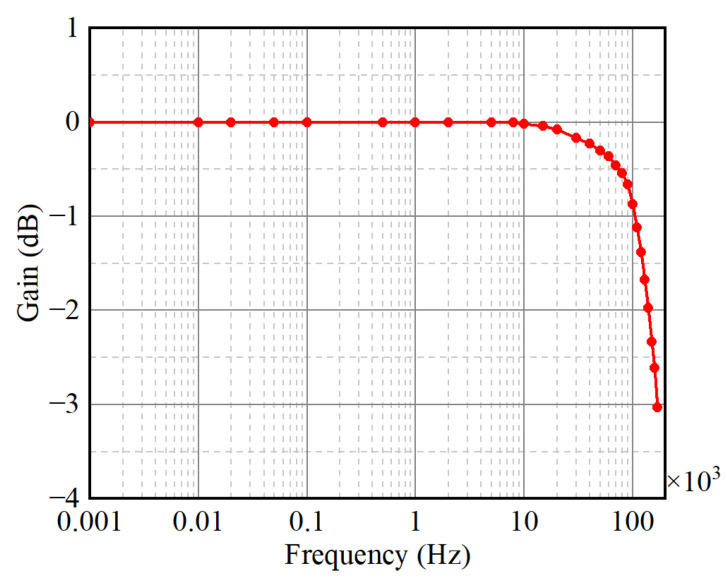
Small-signal bandwidth of the proposed current sensor.

The small-signal bandwidth is measured when the primary current is 5 A. In the experiment, a 0.5 Vrms sinusoid signal is generated by a Rigol DG4102 function waveform generator with a 10 W amplifier. The primary winding with 10 turns wound around the current sensor to achieve an equivalent 5 Arms current is connected to a sampling resistor. The ratio in the form of dB between the voltage of the proposed sensor and the voltage on the sampling resistor is a function of the frequency, as shown in Figure 14. It can be seen that the −1 dB and −3 dB bandwidths of the proposed current sensor are about 100 kHz and 170 kHz, respectively.

### 4.3. Output Noise

**Figure 15:**
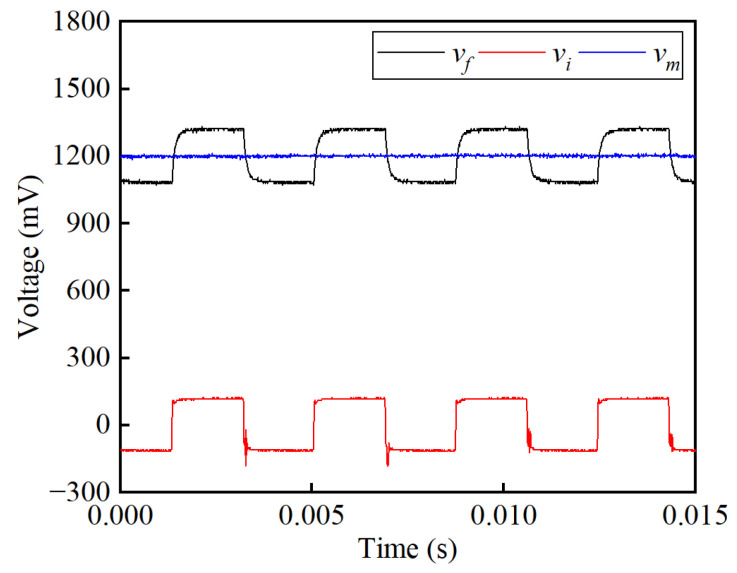
Noise suppression effect of the proposed sensor when the primary current *I_p_* is set to 600 A.

**Figure 16:**
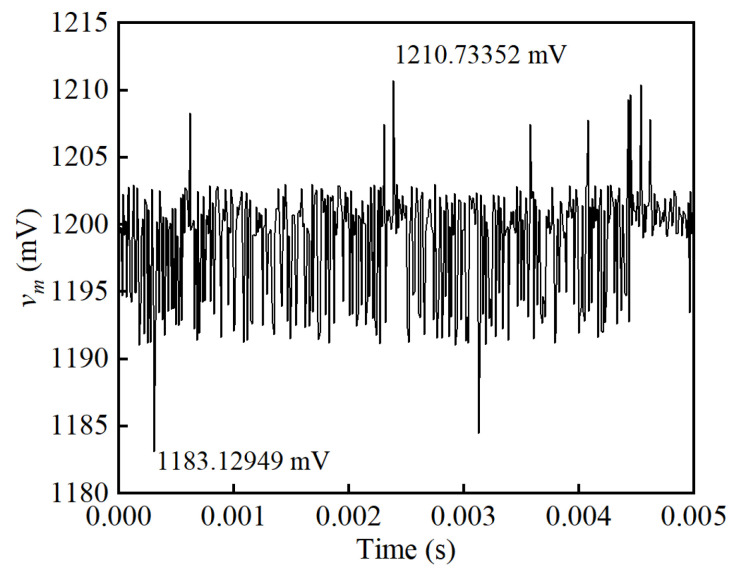
Residual ripple in the output signal *v_m_* when the primary current *I_p_* is set to 600 A.

The voltage *v_f_* on the potential terminal of the feedback resistor *R_f_*, the voltage *v_i_* on the potential terminal of the induction resistor *R_i_*, and the voltage *v_m_* on the potential terminal of the output resistor *R_m_* are obtained to investigate the effectiveness of the proposed noise suppression circuit, as shown in Figure 15. A smooth DC waveform of *v_m_* is obtained after implementing the subtraction circuit and LPF. Figure 16 shows the enlarged waveform to analyze the residual ripple in the output signal *v_m_* when the primary current *I_p_* is set to 600 A. It can be seen that the Max. value is 1210.73 mV, the minimum (Min.) value is 1183.13 mV, and the RMS value is 1199.39 mV. By calculating the power of the signal and its noise, the SNR is obtained as 48.88 dB. In Figure 15, the noise in signal *v_f_* is nearly a square wave with a frequency of 270 Hz, which is the same as the self-oscillating frequency. As expected, a good amplitude and phase consistency is achieved between *v_f_* and *v_i_*. There is a difference in the time constant between the feedback winding *W_f_* and the induction winding *W_i_*, resulting in dynamic deviation in the rise and fall edges. The peak-to-peak value of *v_f_* is 253.09 mV and the RMS value is 1202.07 mV, resulting in an SNR of 19.55 dB. Thus, the SNR is improved from 19.55 dB to 48.88 dB, verifying the effectiveness of the noise suppression.

### 4.4. Power-On Repeatability

**Figure 17:**
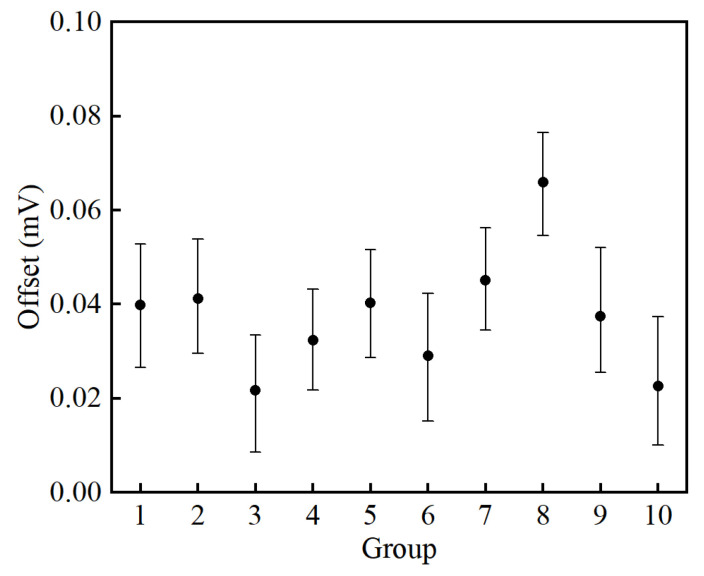
Power-on repeatability of the proposed current sensor.

The memory effect is the cause of zero offsets in DC current sensors [21]. The remanence inside the magnetic core leads to a different working point when the sensor is turned on. Moreover, there are energy storage components such as capacitors and inductors, whose discharge can also cause zero offsets in the feedback signal. Ten groups of measurements of the output signal offset have been carried out to evaluate the power-on repeatability of the proposed sensor. The proposed sensor is thoroughly cut off for 30 min before each measurement so that the residual magnetism of the magnetic core and energy storage of the capacitances and inductances can be reduced to zero as much as possible. Then, the sensor is turned on and 100 values of the output signal *vm* are measured as one group for statistical analysis. In this manner, the offset voltage is measured by a Fluke 289C on the 50 mV range with a resolution of 0.001 mV and an accuracy of 0.05% + 20 to guarantee enough resolution. Results of the signal *v_m_* are converted into current values and presented in Figure 17. The relative standard deviation of the ten groups is 11.66 ppm, indicating a rather low memory effect of the proposed sensor.

### 4.5. Comparison Results

To compare the performance benefits of the proposed one-core self-oscillating fluxgate-based sensor with the multi-core structure, another prototype based on a three-core-four-winding structure in the literature [18], noted as sensor *B*, is designed for MCCB application and fabricated for a contrast test. In order to fairly evaluate the advantages and disadvantages of the two sensors, the magnetic and circuit parameters are designed as similar as possible. We choose the same material models and fabricate the same dimensions for the three cores in sensor *B*. The turn numbers of the exciting, reversing, and compensating windings are equal to *N_ex_*, and the turn number of the feedback winding of Sensor *B* is equal to *N_f_*. To study the characteristics of the proposed sensor extensively, the dimension difference and the comparison characteristics between the proposed sensor and other representative sensors (sensor *B* for multi-core self-oscillating fluxgate, Danisense DS400ID for traditional fluxgate with modulation circuit, and LEM LF 505-S for hall-based sensor) are listed in Table 3.

Compared to the hall-based sensor, the fluxgate-based sensors have higher accuracy. By comparing the proposed sensor, sensor *B*, and Danisense DS400ID, it can be seen that the self-oscillating method benefits from great volume reduction compared to the traditional modulation method, leading to a better application prospect of integrated current detection. Due to the less magnetic core and winding, the volume and cost of the proposed sensor are further reduced by 44.4% and 23.6% compared with sensor *B,* with the multi-core structure, respectively. Despite the relatively simple structure and circuits, the linearity and relative errors are only 0.06 and 0.05 percentage points higher for the proposed sensor compared to sensor *B*. Since the installation space for each sensor probe in the MCCB is 48 × 48 × 30 mm (length × width × height), the small accuracy loss is acceptable with a great reduction of the volume and cost for distribution system applications. In comparison of the small-signal bandwidth, Danisense DS400ID gains a wider bandwidth due to the much higher excitation frequency. In terms of noise suppression, the proposed sensor unexpectedly achieves a higher SNR under 600 A. According to subsequent investigation, the main reason for the lower SNR in sensor *B* is the inconsistency in the bi-directional magnetic cores, resulting in an alternating signal with a frequency similar to the exciting current. These data indicate that the higher manufacturing process requirements will also limit the applications of the multi-core fluxgate sensors. Although the power-on repeatability of the proposed sensor has been slightly improved compared to sensor B due to less energy storage electronics, it is still slightly lower than the Danisense product.

## 5. Conclusions

This paper proposes a new self-oscillating fluxgate-based current sensor with a scheme of one magnetic core and three windings for DC distribution system applications. An example design is carried out for MCCBs with a rated current of 400 A. The characteristics of linearity, small-signal bandwidth, output noise, and power-on repeatability are presented and discussed in this study. The experimental results show that the accuracy of the proposed sensor is 0.15% within the range of 0–600 A. The additional induction winding with the noise suppression circuit has been experimentally verified to improve the SNR from 19.55 dB to 48.88 dB when the primary current is 600 A. Compared with another prototype based on a three-core-four-winding structure, the proposed sensor reduces the volume by 44.4% and the cost by 23.6%, with a relatively simple structure and circuits, while achieving the same level of performance. Further work needs to be carried out to investigate the influence of dynamic electronic performance on induction ripples, the influence of core saturation and thermal effects on high-current detection with a more accurate magnetic core model, and the characteristics in harsh industrial environments.

## Figures and Tables

**Table 1:** Features of different current detection methods.

Method	Bandwidth	Accuracy	Temperature Drift	Range	Power Dissipation
Shunt	kHz-MHz	0.1–2%	25–300 ppm/K	A-kA	W-kW
Hall effect	kHz	0.5–5%	50–1000 ppm/K	A-kA	mW
Giant magnetoresistance	kHz	1–10%	200–1000 ppm/K	mA-kA	mW
Tunnel magnetoresistance	kHz	0.05–3%	50–200 ppm/K	mA-kA	mW
Magneto-optic	kHz-MHz	0.1–1%	<100 ppm/K	kA-MA	W
Fluxgate	kHz	0.001–0.5%	<50 ppm/K	mA-kA	mW-W

**Table 2:** Design of the magnetic parameters.

Parameter	Description	Value	Unit
*μ* *_m_*	Max. magnetic permeability	84,670	
*B_s_*	Saturation magnetic flux density	1.069	T
*H_c_*	Coercivity	0.8488	A/m
*d_o_*	External diameter of the magnetic core	38.18	mm
*d_i_*	Internal diameter of the magnetic core	29.10	mm
*h_c_*	Height of the magnetic core	8.22	mm
*l_c_*	Effective magnetic path length	78.75	mm
*N_ex_*	Exciting winding turns	225	
*N_f_*	Feedback winding turns	500	
*N_i_*	Induction winding turns	500	

**Table 3:** Comparison results between the proposed sensor and other sensors.

Parameter	Proposed	Sensor *B*	Danisense DS400ID	LEM LF 505-S	Unit
Measurement principle	One-core self-oscillating fluxgate	Three-core self-oscillating fluxgate	One-core fluxgate with modulation circuit	Hall effect	-
Nominal current	600	600	600	500	A
External diameter	43.81	45.79	91.5	65	mm
Internal diameter	23.37	23.11	27.6	30.2	mm
Height	20.54	32.54	44.5	31	mm
Volume	2.22 × 10^4^	3.99 × 10^4^	2.66 × 10^5^	8.07 × 10^4^	mm^3^
Cost/Price	41.38	54.13	~400	~500	CNY
Current transfer ratio	500:1	500:1	2000:1	5000:1	-
Excitation frequency	0.27	0.27	31.25	-	kHz
Linearity error	0.07	0.01	0.0001	0.1	%
Relative error	0.15	0.10	0.04	0.6	%
Small-signal bandwidth	100 (−1 dB) 170 (−3 dB)	100 (−1 dB) 170 (−3 dB)	300 (−3.5 dB)	100 (−1 dB)	kHz
SNR at nominal current	48.88	37.66	-	-	dB
Power-on repeatability	11.66	18.83	9	-	ppm
Power consumption at nominal current	7.1	8.5	6	2.23	W

## Data Availability

The data are contained within the article.

## Abbreviations

The following abbreviations are used in this manuscript:

SNR	Singal-to-noise ratio
LPF	Low pass filter
PI	Proportional-integral
PA	Power amplifier
DMM	Digital multimeter
MCCB	Molded case circuit breaker
RMS	Root mean square

## References

[1] Kebede A.A., Kalogiannis T., Van Mierlo J., Berecibar M. (2022). A Comprehensive Review of Stationary Energy Storage Devices for Large Scale Renewable Energy Sources Grid Integration. Renew. Sustain. Energy Rev..

[2] Li Y.Z., Yan Y.F., Chen Y.W., Zhu S.P., Li X.F. (2025). Resistive Onset Determination of Coated Condutors Utilizing a Shunt Current Instead of Voltage Measurement. IEEE Trans. Appl. Supercond..

[3] Liu P., Wang W., Zhou L., Zhao M.C., Wu C.H., He L., Liu H.Y. (2025). Development of a Zero-Flux Hall Current Sensor Dedicated for Large-Current Measurement of Superconducting Cable. IEEE Trans. Appl. Supercond..

[4] Younis M., Abdullah M., Dai S.C., Iqbal M.A., Tang W., Sohail M.T., Atiq S., Chang H.X., Zeng Y.J. (2025). Magnetoresistance in 2D Magnetic Materials: From Fundamentals to Applications. Adv. Funct. Mater..

[5] Wu X., Huang H.H., Dou S., Peng L. (2024). Research on Magnetically Balanced High-Current TMR Sensor for EAST Poloidal Field Power Supply. IEEE Trans. Magn..

[6] Dyankov G., Kolev P., Eftimov T.A., Hikova E.O., Kisov H. (2025). Channeled Polarimetry for Magnetic Field/Current Detection. Sensors.

[7] Garcha P., Schaffer V., Haroun B., Ramaswamy S., Wieser J., Lang J., Chandraksan A. (2022). A Duty-Cycled Integrated-Fluxgate Magnetometer for Current Sensing. IEEE J. Solid-State Circuits.

[8] Cao J., Zhao J., Cheng S. (2019). Research on the Simplified Direct-Current Fluxgate Sensor and its Demodulation. Meas. Sci. Technol..

[9] Ripka P. (2010). Electric Current Sensors: A Review. Meas. Sci. Technol..

[10] Yang X., Wen J., Chen M., Gao Z., Xi L., Li Y. (2020). Analysis and Design of a Self-Oscillating Bidirectionally Saturated Fluxgate Current Sensor. Measurement.

[11] Wei Y., Li C., Zhao W., Xue M., Cao B., Chu X., Ye C. (2022). Electrical Compensation for Magnetization Distortion of Magnetic Fluxgate Current Sensor. IEEE Trans. Instrum. Meas..

[12] Xiao X., Song H., Li H. (2022). A High Accuracy AC plus DC Current Transducer for Calibration. Sensors.

[13] Ponjavic M., Veinovic S. (2021). Low-Power Self-Oscillating Fluxgate Current Sensor Based on Mn-Zn Ferrite Cores. J. Magn. Magn. Mater..

[14] Ding Z., Wang J., Li C., Wang K., Shao H. (2023). A Wideband Closed-Loop Residual Current Sensor Based on Self-Oscillating Fluxgate. IEEE Access.

[15] Yang X., Chen M., Jia Z. (2021). Analysis and Design of a Self-Oscillating Quasi-Digital Fluxgate Current Sensor for DC Current Measurement. Rev. Sci. Instrum..

[16] Ponjavic M.M., Duric R.M. (2007). Nonlinear Modeling of the Self-Oscillating Fluxgate Current Sensor. IEEE Sens. J..

[17] Wang N., Zhang Z., Li Z., Zhang Y., He Q., Han B., Lu Y. (2015). Self-Oscillating Fluxgate-Based Quasi-Digital Sensor for DC High-Current Measurement. IEEE Trans. Instrum. Meas..

[18] Wang N., Zhang Z., Li Z., He Q., Lin F., Lu Y. (2016). Design and Characterization of a Low-Cost Self-Oscillating Fluxgate Transducer for Precision Measurement of High-Current. IEEE Sens. J..

[19] Li J., Ren W., Luo Y., Zhang X., Liu X., Zhang X. (2024). Design of Fluxgate Current Sensor Based on Magnetization Residence Times and Neural Networks. Sensors.

[20] Zhang S., Wang Y., Xie J., Ding T., Han X. (2022). A New Approach for Solving the False Balance of a Closed-Loop Fluxgate Current Transducer. IEEE Trans. Ind. Electron..

[21] Kusters N.L., Moore W.J.M., Miljanic P.N. (2013). A Current Comparator for the Precision Measurement of D-C Ratios. IEEE Trans. Commun. Electron..

